# The role of school connectedness in the prevention of youth depression and anxiety: a systematic review with youth consultation

**DOI:** 10.1186/s12889-022-14364-6

**Published:** 2022-11-25

**Authors:** Monika Raniti, Divyangana Rakesh, George C. Patton, Susan M. Sawyer

**Affiliations:** 1 Centre for Adolescent Health, Murdoch Children’s Research Institute and Royal Children’s Hospital, 50 Flemington Road, Parkville, VIC 3052 Australia; 2 Department of Paediatrics, Melbourne Medical School, University of Melbourne, Royal Children’s Hospital, 50 Flemington Road, Parkville, VIC 3010 Australia; 3 Melbourne Neuropsychiatry Centre, Department of Psychiatry, University of Melbourne and Melbourne Health, 161 Barry Street, Carlton, VIC 3053 Australia

**Keywords:** Schools, Mental health, Adolescents, Young people, Belonging, Intervention, Health promotion

## Abstract

**Background:**

School connectedness reflects the quality of students’ engagement with peers, teachers, and learning in the school environment. It has attracted attention from both the health and education sectors as a potentially modifiable protective factor for common mental health problems. However, the extent to which school connectedness may prevent the onset of youth depression or anxiety or promote their remission is unclear. This systematic review examined evidence for prospective relationships between school connectedness and depression and anxiety, and the effect of interventions to improve school connectedness on depression and anxiety.

**Methods:**

We searched MEDLINE, PsycINFO, PubMed, and ERIC electronic databases for peer-reviewed quantitative longitudinal, or intervention studies published from 2011–21 in English examining relationships between school connectedness and anxiety and/or depression. Participants were 14–24 years old when depression and anxiety outcomes were assessed in any education setting in any country. We partnered with five youth advisers (aged 16–21 years) with lived experience of mental health problems and/or the schooling system in Australia, Indonesia, and the Philippines to ensure that youth perspectives informed the review.

**Results:**

Our search identified 3552 unique records from which 34 longitudinal and 2 intervention studies were ultimately included. Studies were primarily from the United States of America (69.4%). Depression and anxiety outcomes were first measured at 14 years old, on average. Most studies found a significant protective relationship between higher levels of school connectedness and depressive and/or anxiety symptoms; more measured depression than anxiety. A few studies found a non-significant relationship. Both intervention studies designed to increase school connectedness improved depression, one through improvements in self-esteem and one through improvements in relationships at school.

**Conclusions:**

These findings suggest that school connectedness may be a novel target for the prevention of depression and anxiety. We were not able to determine whether improving school connectedness promotes remission in young people already experiencing depression and anxiety. More studies examining anxiety, diagnostic outcomes, and beyond North America are warranted, as well as intervention trials.

**Trial registration:**

PROSPERO 2021 CRD42021270967.

**Supplementary Information:**

The online version contains supplementary material available at 10.1186/s12889-022-14364-6.

## Background

Depression and anxiety are estimated to affect up to one in four young people, with evidence of increasing prevalence in recent years [1]. While improving access to effective treatment is important, prevention is essential to reliably reduce the incidence and associated individual, societal and economic burden of depression and anxiety [2]. Prevention approaches for youth depression and anxiety have commonly focussed on schools, viewing the school curriculum as a platform for effectively delivering a low-dose of individually oriented interventions, typically based on cognitive and behavioural principles [3]. Yet overall these interventions have small effects that are not sustained over time, without evidence of reducing the incidence of depressive and anxiety disorders and with limited scalability [3]. Further, interventions delivered by the education sector (e.g., focused on improving social-emotional learning, learning engagement) have historically neglected mental health outcomes [4]. Rather than further studies with a primary focus on individual factors (e.g., negative thoughts), novel approaches to prevention that recognise schools as social environments that focus on learning, and consider risks and associated strategies for mental health interventions associated with whole-school environments are urgently needed.

Schools are an important resource for influencing the mental health of young people. Most young people are enrolled in schooling, with increasing time spent in secondary and tertiary education [5, 6]. This includes adolescents in low- and-middle income countries (LMICs) who are spending more years in secondary schooling [5, 6]. The most important relationships outside family are often in school and time in education is associated with beneficial life outcomes [7]. The influence of schools on mental health extends beyond developing mental health literacy and the delivery of mental health services, to include the development of social-emotional skills, provision of safe and inclusive environments, and providing a sense of community and support for students, parents and families [8]. In contrast, experiences such as being bullied, disengagement from learning, school dropout, and poor school transitions (e.g., primary to secondary school) have been linked to poorer mental health and social connections in young people [9–12].

School connectedness is a multifactorial construct that includes students’ thoughts (e.g., perceptions of the quality of relationships with teachers and peers and levels of support; example item: *Your teachers care about you*), feelings (e.g., around acceptance, inclusion and belonging, of valuing and enjoying schooling; *I can really be myself at my school*), and behaviours (e.g., participation and engagement in school activities and learning tasks; *You try hard at school*) towards the school environment and learning experiences [13, 14]. This can be towards the school as an institution or community (e.g., *You feel like you are a part of the school*, *I am interested in talking about ways to improve my school*) and/or specific one-to-one social interactions within the school (e.g., *I feel that I can talk to my friends about my problems*, *There is a teacher or some other adult who really cares about me at my school*) [13, 15, 16].

School connectedness is associated with greater academic achievement and psychological wellbeing [17]. Cross-sectional studies link school connectedness with less anxiety, depression, and suicidal thoughts and behaviours, especially for LGBTQ + youth [18–20]. Accumulating evidence also suggests that interventions designed to broadly enhance a school’s social-emotional environment are beneficial for student wellbeing and behavioural outcomes [21–23]. Previous systematic reviews examining similar constructs such as school belonging [24] or school climate [18, 19] (of which school connectedness is one component) on psychological wellbeing and mental health in young people have largely identified cross-sectional studies and failed to differentiate these from longitudinal findings or to examine effects specifically for depression and anxiety. Therefore, the extent to which school connectedness may prevent the onset or promote the remission of depression and anxiety, and the underlying mechanisms of this association, are unclear. For example, schools may be a source of emotional and social support (typically more available to students who experience good connection to school). Greater connection to school might also bring greater learning of cognitive, social, and emotional skills that promote good mental health, or avoidance of hazards to mental health which arise from dropping out of education (i.e., protective relationship). Conversely, connection to school might be associated with academic pressures and in turn, lead to anxiety and poorer mental health (i.e., risk relationship).

To this end, we conducted a systematic review of the evidence for 1) the prospective relationships between school connectedness and depression and anxiety, and 2) the effect of interventions designed to improve school connectedness on depression and anxiety, in young people aged 14 to 24 years.

## Methods

This review was conducted between June and November 2021 as part of the Wellcome Trust’s Commission on “Active Ingredients for Anxiety and Depression in Young People”, in partnership with a youth advisory committee. The review protocol was registered on PROSPERO (CRD42021270967). Cross-sectional studies were included in the original protocol due to uncertainty about how many longitudinal studies were available. In the final review, we excluded cross-sectional studies as a sufficient number of longitudinal studies were identified that better enabled us to answer our research questions. Ethical approval was not required because all data were obtained from published, peer-reviewed journal articles.

### Information sources and search strategy

We searched for articles using MEDLINE, PsycINFO, PubMed and ERIC electronic databases on July 12^th^ 2021, using free-text and controlled terms related to the concepts of: 1) school connectedness; 2) depression and anxiety; 3) youth. The MEDLINE search strategy, used as the basis of the search for the other databases, is shown in Additional File 1.

### Eligibility criteria

We included peer-reviewed journal articles published in English from January 1^st^ 2011 to July 12^th^ 2021, as previous reviews have shown that very few longitudinal and intervention studies examining school connectedness and mental health outcomes were published prior to 2011 [19]. We included quantitative observational (longitudinal) and intervention studies of any design. No other restrictions were applied.

#### Participants

Participants were adolescents and young people aged 14 to 24 years (the age range was set by the funder) at the time that depression and anxiety outcomes were measured, and attending a primary/elementary, secondary or tertiary/further education setting in any country. A study that spanned a wider age range was included if the mean age lay within or very close to our specified age range or where results were presented separately for the age range of interest. Participants could be from any population (e.g., clinical, community).

#### Exposure/intervention

To be eligible for inclusion, longitudinal studies had to examine the relationship between school connectedness and later anxiety and/or depression. We included studies that measured one or more component of school connectedness. We also included studies that used different terminology such as ‘school belonging’ or ‘school climate’ when it was clear that the construct was synonymous with our definition of school connectedness, where the study reported on the sub-construct of ‘school connectedness’ separately, or where an established measure of school connectedness was used (e.g., Psychological Sense of School Membership Scale [25], School Connectedness Scale [26]) [14]. Intervention studies needed to evaluate the effect of an intervention designed to improve school connectedness that was delivered within a school setting. We kept our definition of an intervention broad to capture the breadth of possible components within whole-school approaches [8], for example, the delivery of a discrete education program, school curriculum or policy change, changes to a school’s social-emotional or physical environment, or school staff professional development training.

#### Outcomes

We defined anxiety and/or depression as any combination of thoughts, feelings, and behaviours associated with depression and anxiety (e.g., maladaptive thoughts, enduring sadness, sudden panic, sleeping difficulties) across the continuum of experience that are persistent, pervasive and cause difficulties in daily life (i.e., not general psychological wellbeing or transient emotional responses). To be included, studies needed to examine prospective associations between school connectedness and anxiety and/or depression score (or similar such an ‘internalising symptoms’) or diagnostic status over time, or before and after an intervention.

### Selection process

Article deduplication and title, abstract and full text screening were conducted in Covidence software by a single researcher. Eligibility criteria were discussed with the research team when required. A second researcher independently screened full-text articles where eligibility was unclear, with any discrepancies resolved through discussion with the research team.

### Data collection process and data items

Data were extracted by a single researcher into an Excel database who engaged closely with a second researcher and the research team when clarification was required. Extracted data included study sample size, country of origin, study design, recruitment and sampling method, participant characteristics, exposure and outcome measures, intervention characteristics, time between data collection points, participant loss to follow-up, confounders, and relevant findings (e.g., direction of association, effect sizes where possible).

### Study risk of bias assessment

Study quality assessment was conducted independently by one researcher with extensive experience in conducting study quality assessments and checked by a second researcher using National Institute of Health (NIH) tools appropriate for the study design [27], namely the Quality assessment tool for observational and cross-sectional studies or the Quality assessment of controlled intervention studies tool. Both researchers met to clarify ratings and consulted with the other co-authors to reach a consensus rating where required. Studies were assessed on 14 criteria and rated ‘good’, ‘fair’ or ‘poor’ quality per NIH guidance.

### Synthesis methods

Data were synthesised using narrative synthesis and summary tables, with results for longitudinal and interventions studies presented separately. A study’s primary findings were classified as being ‘protective’, ‘risk’ or ‘not significant’ for prospective relationships between school connectedness and depression and anxiety, noting where studies had mixed findings. We considered the generalisability of the results across subgroups (e.g., by sex/gender) and moderators and mediators of effects. Due to heterogeneity across studies in terms of how school connectedness and depression and anxiety were measured, and the types of statistics reported, it was not possible to evaluate overall effect sizes using meta-analyses or compare effect sizes across studies.

### Partnership with youth advisory committee

We partnered with a committee of five youth advisers (age range 16 to 21 years) with lived experience of mental health problems and/or the schooling system, located in Australia, Indonesia, and the Philippines. The primary role of the advisers was to ensure that youth perspectives informed the interpretation and dissemination of the findings and future directions for research and practice. Youth advisers were not involved in article screening, data extraction, or study quality assessments. Advisers were recruited using our institutional social media channels and professional networks. Consistent with youth advisory practices in research, the formation of the youth advisory committee was exempt from ethical review as advisers were expert consultants rather than research participants [28, 29]. The research team and youth advisers engaged in three consultation meetings (September to November 2021) via Zoom. Youth advisers also reviewed documents and provided input outside of meetings. Youth advisers were financially reimbursed for their time.

## Results

**Fig. 1:**
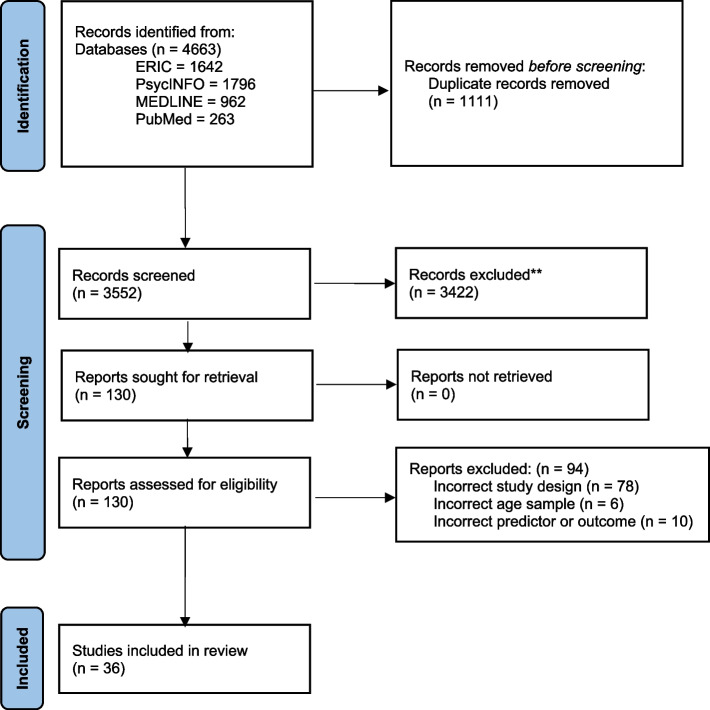
PRISMA flowchart of search results at each step of the systematic review

We identified 3552 unique records in our search which ultimately yielded 36 articles that were included in the review (Fig. 1). Four studies used data from the National Survey of Child and Adolescent Well-Being (NSCAW) [30–33] and four studies used data from the National Longitudinal Study of Adolescent to Adult Health (Add Health) [34–37]. Three studies appeared to meet inclusion criteria but were ultimately excluded because they examined suicide attempts and not depression or anxiety (*n* = 2) [38, 39] or used cross-sectional data in analyses (*n *= 1) [40].

### Study characteristics

The study characteristics of the 36 included articles (34 longitudinal and 2 intervention) are presented in Tables 1 and 2. For longitudinal studies, sample sizes ranged from 119 to 20,745 participants. The intervention studies had sample sizes of 497 [41] and 5539 [42]. Across all included studies, the average participant age at baseline ranged from 10 to 19 years old, although some studies only reported participants’ school grade and not age. The most common average baseline age was 12–13 years old (when school connectedness was measured) and the most common average age when depression and anxiety outcomes were first measured was 14 years old. Around a third of studies (*n* = 12) [15, 35, 37, 43–51] included baseline and follow-up assessments only. Eleven studies [32, 33, 52–60] included three timepoints of data, 10 studies [16, 30, 31, 34, 36, 61–65] included four timepoints and one study [66] included five timepoints. The study [62] with the longest period of data collection followed participants with an average age of 16 years at baseline until they were 43 years old.

**Table 1:** Study characteristics for longitudinal studies presented by exposure (school connectedness and school disconnectedness constructs)

**Author (Year)**	**N (% female), group characteristics, county**	Mean age (years) ± SD (or range)*	**School connectedness measure**	**Depression and anxiety measure**	**Relevant findings**				
					**Exposure**	**Outcome**		**Direction of effect**	**Additional information**
**School Connectedness**									
Arango et al. (2018) [43]	142 (75%), USA	T1: 13.41 (1.12) T2: 6 months later	School Connectedness Scale	RADS-2:SF	School connectedness (T1)	Depression (T2)		Protective	
Arora et al. (2017) [15]	186 (49%), Asian American youth, USA	T1: 12.50 (1.16) T2: 1 yr later	School engagement (5 items), Teacher support (5 items)	CESD (adapted), State–Trait Anxiety Inventory for Children (adapted)	Teacher support (T1)	Depression (T2)		Protective	When teacher support was moderate-to-high at T1, high levels of anxiety at T1 were associated with increased levels of depressive symptoms at T2, an association that was not present under conditions of low teacher support
					School engagement (T1)	Depression (T2)		NS	
Davis et al. (2019) [61]	2,177 (48%), USA	T1: 12.3 (0.7) T2: NR T3: NR T4: 13.8 (0.72)	PSSM (4/20 items)	Orpinas Modified Depression Scale	School belonging (T1)	Depression (T2)		NS	Result across the whole sample
					School belonging (T2)	Depression (T3)		NS	Result across the whole sample
					School belonging (T3)	Depression (T4)		NS	Result across the whole sample
					School belonging (T1)	Depression (T2)		Protective	Result for females only
					School belonging (T2)	Depression (T3)		Protective	Result for females only
					School belonging (T3)	Depression (T4)		Protective	Result for females only
DeWit et al. (2011) [53]	2,616 (54%), Canada	T1: 13.77 (0.54) T2: ~ 6 months later T3: 1 yr later	Social Support Appraisals Scale (SSAS) of the Survey of Children’s Social Support	CESD; Generalized Social Avoidance and Distress subscale of the Revised Social Anxiety Scale for Children	Classmate support (slope)	Depression (slope)		Protective	
					Teacher support (slope)	Depression (slope)		Protective	
					Classmate support (intercept)	Depression (slope)		NS	
					Teacher support (intercept)	Depression (slope)		NS	
					Classmate support (slope)	Social anxiety (slope)		Protective	
					Teacher support (slope)	Social anxiety (slope)		Protective	
					Classmate support (intercept)	Social anxiety (slope)		Risk	
					Teacher support (intercept)	Social anxiety (slope)		Risk	
Fulco et al. (2019) [16]	427 (50%), USA	T1: 14 T2: 15 T3: 16 T4: 17	School engagement (9 items)	CESD (13 items)	Change in school engagement (T1 to T4, time-varying covariate)	Change in depressive symptoms (T1 to T4, non-significant)		Protective	Result for males only
					Change in school engagement (T1 to T4, time-varying covariate)	Change in depressive symptoms (T1 to T4, linear growth)		Protective	Result for females only
Gonzales et al. (2014)** [54]	516 (51%), Mexican American adolescents, USA	T1: 12.3 (0.54) T2: 2 yrs later T3: 5 yrs later	School Engagement Scale—draws items from The School is Important Now Scale, the Academic Liking Scale, and the Importance of Education Scale	YSR at T1, T2, ASR at T3	School engagement (T2)	Internalizing problems (T3)		Protective	T2 school engagement mediated the association between a family focused intervention and T3 internalising problems
Hatchel et al. (2018) [55]	404 (45.3% F; 51.8% M, 2.9% other), LGBTQ youth, USA	T1: 15.27 (15–17) T2: 1 yr later T3: 2 yrs later	PSSM (9 items)	Orpinas Modified Depression Scale (9 items)	School belonging (T1)	Depression (T2)		Protective	School belonging mediated the relationship between victimization and depression
					School belonging (T2)	Depression (T3)		Protective	
Jiang et al. (2020) [45]	2,041 (46%), Migrant adolescents, China	T1 13.6 (0.71) T2: 1 yr later	Emotional engagement (5 items)	CESD (5 items)	Emotional engagement (T1)	Depression (T2)		Protective	Emotional school engagement partially mediated the relationship between teacher discrimination and depression
Joyce (2019) [35]	13,120 (52%), USA	T1: Grade 7–12 T2: 1 yr later	Teacher support (2 items)	CESD (adapted)	Getting along with teachers (T1)	Depression (T2)		Protective	School connectedness at T2 partially mediated the effect between 1) getting along with teachers at T1 and depression at T2 and 2) feeling cared for by teachers at T1 and depression at T2
					Feeling cared about by teachers (T1)	Depression (T2)		Protective	
Klinck et al. (2020) [46]	1,344 (51%), USA	T1: 12.73 (11–14) T2: ~ 6 months later	School Connectedness Scale	CESD; SCARED (total score and subscales)	School connectedness (T1)	Depression (T2)		Protective	
					School connectedness (T1)	Anxiety (T2)		NS	
					School connectedness (T1)	School avoidance (T2)		Protective	Association was not significant for other SCARED subscales (GAD, PD, SAD, SEP)
					Moderation analyses: Anxiety: Anxiety moderated the association between school connectedness and depression such that in adolescents at low risk of an anxiety disorder, higher school connectedness at T1 predicted lower levels of depressive symptoms at T2. Conversely, in adolescents at high risk of an anxiety disorder, there were no significant relationships between school connectedness and depressive symptoms. Gender: Time 1 associations between school connectedness and internalizing problems were stronger in magnitude for girls as compared with boys across all models. Race: In addition, race moderated the association, such that in adolescents identifying as non-Hispanic White, Hispanic, or Latinx, higher levels of school connectedness at T1 was associated with lower depression at T2, which was not the case for adolescents identifying as Black/African American				
Leonard et al. (2016) [30]	769 (56%), Children in contact with CWS, USA	T1: 12.69 (1.3) T4: 3 yrs later	11 items from Drug-Free Schools and Communities Act Survey	YSR	School engagement (T1)	Internalizing problems (T4)	Protective		School engagement did not moderate the association between placement instability and internalizing problems
Leonard & Gudiño (2016) [31]	224 (58%), Children in out-of-home care during the study period, USA	T1: 12.85 (1.25) T4: 3 yrs later	11 items from Drug-Free Schools and Communities Act Survey	YSR	School engagement (T1)	Internalizing problems (T4)	NS		School instability prospectively predicted internalizing symptoms
Lester et al. (2013) [63]	T1: 1,054 (~ 54%) T2: 1,743 (additional students in secondary school [grade 8] that were not enrolled in the primary school at T1 [grade 7]), Australia	T1: ~ 12 (end of grade 7) T2: ~ 12 (start of grade 8) T3: ~ 13 T4: ~ 14	School Connectedness Scale (4 items)	DASS-21	School connectedness (T1)	Depression (T2)	NS		Result for males only
					School connectedness (T1)	Depression (T2)	Protective		Result for females only
					School connectedness (T2)	Depression (T3)	Protective		Result for males only
					School connectedness (T2)	Depression (T3)	Protective		Result for females only
					School connectedness (T3)	Depression (T4)	Protective		Result for males only
					School connectedness (T3)	Depression (T4)	NS		Result for females only
					School connectedness (T1)	Anxiety (T2)	Protective		
					School connectedness (T2)	Anxiety (T3)	Protective		
					School connectedness (T3)	Anxiety (T4)	Protective		
Lester & Cross (2015) [56]	1616 (50% F) Australia	T1: 12 T2: 13 T3: 14	Teacher connectedness (Teacher Connectedness Scale), School connectedness (School Connectedness Scale), The peer support at school scale (adapted from the 24-item Perceptions of Peer Social Support Scale)	Emotional symptoms (SDQ), Depression (DASS-21), Anxiety (DASS-21)	School connectedness (T1)	Depression (T2)	Protective		
						Anxiety (T2)	Protective		
						Emotional problems (T2)	Protective		
					Teacher connectedness (T1)	Depression (T2)	NS		
						Anxiety (T2)	NS		
						Emotional problems (T2)	NS		
					Peer support (T1)	Depression (T2)	Protective		
						Anxiety (T2)	Protective		
						Emotional problems (T2)	Protective		
					School connectedness (T2)	Depression (T3)	Protective		
						Anxiety (T3)	Protective		
						Emotional problems (T3)	Protective		
					Teacher connectedness (T2)	Depression (T3)	NS		
						Anxiety (T3)	NS		
						Emotional problems (T3)	NS		
					Peer support (T2)	Depression (T3)	Protective		
						Anxiety (T3)	Protective		
						Emotional problems (T3)	Protective		
Li & Lerner (2011) [64]	1,977 (43%), USA	T1: 11 (0.52) T4: 3 yrs later	Emotional school engagement (3 items)	CESD	Emotional school engagement	Depression	Protective		Four growth trajectories established for emotional school engagement (decreasing, moderate, high with decrease, and highest). Emotional engagement trajectory groups at T1 were associated with T4 depression. Members of the decreasing group of emotional engagement reported the highest levels of depression, whereas youth in the highest group were least depressed. Students who experienced high but decreasing emotional engagement were more depressed than youth in the highest group
Loukas et al. (2016) [57]	296 (50%), USA	T1: 11.7 (0.76) T2: 12.3 (0.49) T3: 13.25 (0.44)	5 items from the National Longitudinal Study of Adolescent Health	CDI	School connectedness (T1)	Depression (T2)	Protective		
					School connectedness (T1)	Depression (T3)	Protective		
					School connectedness (T2)	Depression (T3)	Protective		
Markowitz (2016) [36]	9,698 (53%), USA	T1: 15.76 (1.57) T2: 1 yr later T3: 5 yrs later	6 items	CESD (9 items)	School connection (T2)	Depression (T3)	Protective		There was an interaction between early adversity and school connection such that early adversity was associated with depressive symptoms only for boys with low levels of school connection
McNeil et al. (2020) [32]	627 (53%), Children in contact with CWS, USA	T1: 12.5 (1.13) T2: 1.5 yrs later T3: 3 yrs later	11 items from Drug-Free Schools and Communities Act Survey	CDI	School engagement (T1)	Depression (slope)	NS		
					School engagement (slope)	Depression (slope)	Protective		
					Moderation analyses: Decreasing school engagement explained the association between parental non-involvement and increasing depression symptoms for Hispanic youth, but the indirect effect of parental non-involvement on depressive symptoms via school engagement was negative in White youth (increasing school engagement with low parental involvement led to decreasing depressive symptoms). The indirect effect was not significant for African American or Asian/other participants				
Moffa et al. (2016) [47]	1,867 (51%, 1% other), USA	T1: Grade 9–11 T2: 1 yr later	5 items from the School Satisfaction subscale of the Multidimensional Students’ Life Satisfaction Scale	Internal distress (anxiety and depressive symptoms measured using 7 items)	School belonging (T1)	Internal distress (T2)		Risk	The authors noted that “the explained variance in internal distress was not substantial, Cohen’s f2 = .006. For this observed negligible effect size, the achieved power was not adequate (.75)”. A 1 standard deviation increase in school connectedness only predicted a 0.08 standard deviation increase in internal distress
Okado et al. (2018) [58]	209 (50%; survivors of pediatric cancer), USA	T1: 12.48 (2.86) T2: 13.20 (2.93) T3: 15.64 (2.93)	Hemingway Measure of Adolescent Connectedness	Behavior Assessment System for Children	School connectedness (T2)	Internalizing problems (T3)		Protective	
					Teacher connectedness (T2)	Internalizing problems (T3)		Protective	
					Peer connectedness (T2)	Internalizing problems (T3)		Protective	
Pierre et al. (2020) [48]	119 (0%) African-American males, USA	T1: 15.33 (0.95) T2: 16.56 (0.97)	PSSM	DASS	School belonging (T1)	Depression (T2)		NS	Sample included males only. T1 violence victimization and witnessing violence did not predict T2 depressive, anxiety, or stress symptoms at high levels of school belongingness
					School belonging (T1)	Anxiety (T2)		NS	
					School belonging (T1)	Stress (T2)		NS	
Pössel et al. (2016) [66]	2,545 (51%), Australia	T1: Grade 8 (13.11 [0.56]) T2—T5: Grade 9–12 (1 yr intervals)	Teacher-reported school climate (12 items)—obtained scores of two factors (teacher–student relationships and safe/orderly environment), which were averaged for analyses. The correlation between the two factors was 0.6, *p* < 0.001	CESD	School climate (T1)	Depression (slope T1 to T5)		Risk	No difference between males and females
					School climate (T1 to T5 slope)	Depression (T1 to T5 slope)		NS	
Sanders et al. (2020)**[49]	294 (? F in 7th grade, but 54% at original recruitment in KG), USA	T1: 7th Grade T2: 9th Grade	School bonding and Affiliation with teacher subscales (People in My Life Questionnaire); General Adjustment subscale (SAQ)	SDQ	School bonding	Emotional symptoms		Protective	Estimated latent profiles of change in emotional symptoms and change in school bonding, resulting in three profiles (each) of both variables (high distress, medium distress and low distress for emotional symptoms, and strong school bond, average school bond, and weak school bond for school bonding). The profiles showed a moderate level of intercorrelation (r = 0.41), and 50% of the sample fell into a profile reflecting the same adjustment level (e.g., low, medium, or high) in both domains of emotional symptoms and school bonding
Shochet & Smith (2014) [59]	504 (45%), Australia	T1: 13.3 (0.5) T2: 1 yr later T3: 1.5 yrs later	PSSM	CDI	School connectedness (T1)	Depression (T2)		Protective	
					School connectedness (T1)	Depression (T3)		Protective	
					School connectedness (T2)	Depression (T3)		Protective	Mediated the association between classroom environment and depression
Shochet et al. (2011) [60]	504 (45%), Australia	T1: 13.3 (0.5) T2: 1 yr later T3: 1.5 yrs later	School connectedness subscales: Caring Relations, Acceptance, and Rejection (PSSM)	CDI	Caring Relations (T2)	Depression (T3)		NS	Result for males only
					Acceptance (T2)	Depression (T3)		Protective	Result for males only
					Rejection (T2)	Depression (T3)		NS	Result for males only
					Caring Relations (T2)	Depression (T3)		NS	Result for females only
					Acceptance (T2)	Depression (T3)		Protective	Result for females only
					Rejection (T2)	Depression (T3)		NS	Result for females only
Stiles & Gudiño (2018) [33]	2633 (52%), Youth in contact with CWS, USA	T1: 10.04 (2.72) T2: 1.5 yrs later T3: 3 yrs later	11 items adapted from the Drug-Free Schools and Community Act Survey	CBCL (subscale)	School engagement (T1)	Internalizing problems (T2)		NS	
					School engagement (T2)	Internalizing problems (T3)		NS	
Wright & Wachs (2019) [51]	416 (46%), USA	T1: 13.89 (0.41) T2: 1 yr later	School belongingness (18 items)	CESD; The Multidimensional Anxiety Scale for Children	School belongingness (T1)	Depression (T2)		Protective	For T2 depression and anxiety, there was a significant three-way interaction between cyber victimization, school-belongingness, and ethnicity. T2 depression/anxiety and cyber victimization were more strongly associated at lower levels of school-belongingness for Latinx adolescents
					School belongingness (T1)	Anxiety (T2)		Protective	
Yu et al. (2016) [65]	236 (58%), China	T1: 7th Grade T2: 6 months later T3: 1 yr later T4: 14.34 (0.57); 1.5 yrs later	School Engagement Scale at T2 and T3	YSR at T3 and T4 (mean of 16 items)	School engagement (T2)	Anxiety & Depression (T3)		Protective	
					School engagement (T2)	Anxiety & Depression (T4)		Protective	
					School engagement (T3)	Anxiety & Depression (T4)		Protective	
**School Disconnectedness**									
Benner et al. (2017) [44]	252 (50%), Predominantly Latina/o and African American youth, USA	T1: 14.38 (0.46) T2: 15.58 (0.51)	Gottfredson’s measurement (5 items)	CDI	Decreasing school belonging (T1 to T2) (compared to stable and increasing school belonging)	Change in depressive symptoms (T1 to T2)		Risk	
Boen et al. (2020) [34]	20,475 (?), USA	T1: Grade 7–12 T2: 1/2 yrs later T3: 5/6 yrs later T4: 12/13 yrs later	Component obtained from Principal Component Analysis of interview and questionnaire items	CESD (9 items)	Low school connectedness (T1)	Depression (trajectory T1 to T4)		Risk	Low school connectedness was found to have a strong positive association with depressive risk, that diminished over time
Cristini et al. (2012) [52]	347 (53%), Italy	Data were collected at the end of each of the three middle school years (T1, T2, T3)	Teacher-student and student–student relationships using the School Situation Questionnaire	Depression and anxiety (5 items)	Socially isolated cluster (low on student–student relationships) at T1	Depression/Anxiety (T2 & T3)		Risk (at T2 and T3)	Socially isolated group showed higher levels of emotional problems than the well-adjusted cluster at each wave
Gunnarsódttir et al. (2021) [62]	944 (48%), Sweden	T1: 16 T2: 21 T3: 30 T4: 43	Principal Component Analysis on variables considered to capture interrelations occurring within the family and the school context	Depression (captured using six symptom measures)	Poor school connectedness (T1)	Depression (T2 to T4)		Risk	
Tucker et al. (2011) [50]	4,329 (52%), USA	T1: 14.83 (95% CI 14.82 – 14.85) T2: ~ 21	School disengagement (5 items)	CESD (8 items)	School disengagement (T1)	Depression (T2)		Risk	
Wickrama & Vazsonyi (2011) [37]	20,745 (49%), USA	T1: 13—19 yrs T2: NRT3: 6 yrs later	School disengagement (4 items)	Depression (CESD; 8 items)	School disengagement (T1)	Depression (change in symptoms T1 to T3)		Risk	Interaction effects between race/ethnicity and school disengagement and between school minority concentration and school experiences were also statistically significant. For Hispanic American adolescents, school disengagement had a stronger influence on changes in depressive symptoms than for European American adolescents (reference group)

*NS*  not significant, *School grade reported where age not provided, duration of follow-up timepoint compared to T1 (baseline); **Intervention studies classified as longitudinal for this review as the interventions were not designed to increasing school connectedness, *PSSM*   Psychological sense of school membership scale, *CESD*   Center for Epidemiological Studies – Depression scale, *YSR * Youth Self-Report, *ASR*  Adult Self-Report, *SCARED*  The Screen for Child Anxiety Related Disorders, *DASS-21*  Depression Anxiety and Stress Scale, *SDQ*  Strengths and Difficulties Questionnaire, *CDI*  Children’s Depression Inventory, *CBCL*   Child Behavior Checklist, *RADS-2:SF*  Reynolds Adolescent Depression Scale:Short-Form, *GAD  * Generalised Anxiety Disorder, *PD *  Panic Disorder, *SAD*  Social Anxiety Disorder. *SEP*  Separation Anxiety Disorder, *T*  Time, *CWS*  Child Welfare System

**Table 2:** Study characteristics for intervention studies

**Author (Year)**	**N (% female), group characteristics, country**	**Mean age (years) ± SD (or range)**	**Study design and data collection (pre-post, follow-up)**	**School connectedness, depression and anxiety measures**	**Intervention description (Control condition)**	**Relevant findings**
Blossom et al. (2020) [41]	Intervention: 241 (61.8%),Control: 256 (64.5%), Youth with elevated depression, USA	Intervention at T1 T2: 3 months later (8th grade) T3: 9 months later T4: 12 months later T5: 1.5 yrs later (9th grade)	Randomised controlled trial: 5 waves; Baseline, 3-month follow-up (school attachment), 9-month follow up (self-esteem) and 18-month follow-up (outcomes)	School attachment (High School Questionnaire); Depression (Short Mood and Feelings Questionnaire)	HSTP: aimed to reduce risks of depressive symptoms among students transitioning to high school by increasing self-esteem and school attachment through providing them with social support (both at school and with caregivers) and promoting students’ participation in positive, school-based activities (A one-on-one standardized interview and clinical follow-up with a trained clinician, with a telephone call to parents to review concerns and to make recommendations for additional services as needed)	School attachment at T2 did not mediate the effects of the HSTP intervention on depressive symptoms at T5 (95% CI = 0.03 to 0.04). Sequential mediation model: HSTP intervention influenced school attachment at T2 which contributed to self-esteem at T3, which in turn contributed to lower depressive symptoms at T5 (95% CI = 0.02 to 0.0005). After accounting for self-esteem the direct effects of the intervention and T2 school attachment on T5 depressive symptoms were not significant (B = .02, p = .79). A second mediation model where the HSTP intervention predicted T3 school attachment, which predicted T4 self-esteem, which in turn predicted T5 depression was also significant (95% CI = 0.04 to 0.003)
Singla et al. (2021) [42]	Intervention: 2854 (53%), Control: 2685 (52%), India	Intervention: 13.70 (95% CI = 13.67–13.73) Control: 13.71 (95% CI = 13.68–13.74) T2: 8 months later T3: 17 months later	Subset of larger randomised controlled trial: 3 waves; Baseline, 8-month follow-up, and 17-month follow-up	Relationship to school and school belongingness (subscales of Beyond Blue School Climate Questionnaire); Depression (Patient Health Questionnaire-9)	SEHER: In addition to the information provided to the control arm, the intervention emphasized the importance of a positive school climate (supportive relationships between school community members, a sense of belonging to the school, a participative school environment) by identifying several areas for action (e.g., promoting social skills among adolescents) (Trained teacher in each school who conducted classroom-based sessions on life skills, including developmental changes, developing positive and responsible relationships, gender and sexuality, prevention of HIV and other sexually transmitted infections, and substance use)	Relationships at school 8 months post randomization mediated the association between the intervention and depressive symptoms 17 months post randomization (X- > M: standardized beta = 1.116 [0.20], M—> Y: standardized beta = -0.064 [0.017]). Indirect effect: -0.071 (-0.098 to 0.036). School belongingness 8 months post randomization did not mediate the relationship between intervention status and depressive symptoms 17 months post randomization (X- > M: standardized beta = 0.878 [0.22], M—> Y: standardized beta = 0.029 [0.016]); X- > M (effect of independent variable on mediator) M- > Y (effect of mediator on dependent variable)

Study participants were primarily recruited from middle and secondary schools. No participants were recruited from tertiary or further education settings. One study (intervention study) [41] recruited participants with elevated depressive symptoms, four studies [30–33] recruited young people engaged with the welfare system, five studies [15, 44, 45, 48, 54] were conducted with young people from minority groups, and one study [58] was conducted with paediatric cancer patients. Studies were from seven different countries, with the majority from the United States of America (USA; *n* = 25, 69.4%), followed by Australia (*n* = 5) [56, 59, 60, 63, 66], China (*n* = 2) [45, 65], Canada (*n* = 1) [53], Italy (*n* = 1) [52], India (*n* = 1) [42], and Sweden (*n* = 1) [62].

Studies varied in their conceptualisation and measurement of school connectedness and depression and anxiety.

### How was school connectedness operationalised?

Twenty-two studies [32–34, 36, 37, 41, 43, 45, 46, 48, 51, 52, 54–57, 59, 61–63, 65, 66] examined school connectedness in a multifaceted and holistic manner (e.g., items related to school attachment, engagement and climate totalled to produce an overall score). Around a third of these studies included items that were heavily weighted towards the relational and emotional aspects of school connectedness (e.g., closeness to teachers and peers, sense of belonging, enjoyment of school), whereas other studies included items that also reflect other aspects of school connectedness (e.g., participation and engagement in school activities and learning). Fourteen studies [15, 16, 30, 31, 35, 42, 44, 47, 49, 50, 53, 58–60, 64] only examined specific components of school connectedness (e.g., items reflecting teacher support, classmate support, or school engagement separately), with several of these studies including multiple components within their analysis. One study used teacher-reported school connectedness rather than student-report [66]. The most commonly used measures of school connectedness were the Psychological Sense of School Membership scale (*n* = 5) [48, 55, 59–61], the School Connectedness Scale (*n* = 4) [43, 46, 56, 63], and the School Engagement Scale (*n* = 2) [54, 65]. However, studies did not necessarily use all items from these scales and varied in whether they reported a total score, subscale scores or item scores. Four studies used items from the Drug-Free Schools and Community Act Survey [30–33]. Twelve studies used a single item [35] or a combination of items [15, 16, 34, 36, 37, 45, 50, 57, 62, 64, 66] developed or selected by the researchers in their analyses.

### How were mental health outcomes operationalised?

Thirty-one studies (including the two intervention studies) [15, 16, 32, 34–37, 41–46, 48, 50, 51, 53, 55–57, 59–64, 66] examined depressive symptoms as an outcome, six studies [46, 48, 51, 53, 56, 63] examined anxiety symptoms, and ten studies [30, 31, 33, 47, 49, 52, 54, 56, 58, 65] examined a combination or equivalent (e.g., internalising symptoms). The most common measure of depression was the Center for Epidemiological Studies – Depression scale (CESD; *n* = 13) [15, 16, 34–37, 45, 46, 50, 51, 53, 64, 66] followed by the Children’s Depression Inventory (CDI; *n* = 5) [32, 44, 57, 59, 60] and the Depression Anxiety Stress Scale (DASS-21; *n* = 3) [48, 56, 63]. All except two studies [55, 61] used validated depression and anxiety scales, although several used adapted versions. No study examined the clinical diagnosis of depression or anxiety as an outcome.

### What were the interventions?

One of the intervention studies was conducted in the USA in young people in 8^th^ Grade (*n* = 241 intervention, *n *= 256 control) with elevated levels of depression [41]. That study (‘The High School Transition Program’; HSTP) aimed to reduce the risk of depressive symptoms in students transitioning to high school. The intervention was designed to provide social/school support and encouraged participation in positive school activities in order to improve school attachment and self-esteem. Another intervention study (the ‘Strengthening Evidence base on scHool-based intErventions for pRomoting adolescent health program’; SEHER) was conducted with young people aged around 13.5 years at baseline (*n* = 2854 intervention, *n *= 2685 control) in India [42]. That study aimed to improve depressive symptoms in secondary school students by improving school climate, including by encouraging supportive relationships between members of the school community, promoting school belonging, increasing participation in school activities, and promoting social skills among students.

### Risk of bias

Study quality assessment ratings were completed for the 36 included studies (Tables 3 and 4). Twenty-six studies were rated ‘good’ quality (including the two intervention studies), eight were ‘fair’ quality, and two were rated ‘poor’ quality. Studies often did not control appropriately for confounders in their models (e.g., baseline depressive and anxiety symptoms, sex/gender). Study sample size was rarely justified in the included studies and nearly half of studies did not report the number or characteristics of participants lost to follow-up. Studies varied widely on whether they used exposure and outcome measures that were valid and reliable. However, as the pattern of results remained unchanged when only ‘good’ quality studies were considered, all studies are included in the synthesis below.

**Table 3:** Risk of bias assessment for longitudinal studies

	1	2	3	4	5	6	7	8	9	10	11	12	13	14	Overall
Author (Year)	Was the research question or objective in this paper clearly stated?	Was the study population clearly specified and defined?	Was the participation rate of eligible persons at least 50%?	Were all the subjects selected or recruited from the same or similar populations (including the same time period)? Were inclusion and exclusion criteria for being in the study prespecified and applied uniformly to all participants?	Was a sample size justification, power description, or variance and effect estimates provided?	For the analyses in this paper, were the exposure(s) of interest measured prior to the outcome(s) being measured?	Was the timeframe sufficient so that one could reasonably expect to see an association between exposure and outcome if it existed?	For exposures that can vary in amount or level, did the study examine different levels of the exposure as related to the outcome (e.g., categories of exposure, or exposure measured as continuous variable)?	Were the exposure measures (independent variables) clearly defined, valid, reliable, and implemented consistently across all study participants?	Was the exposure(s) assessed more than once over time?	Were the outcome measures (dependent variables) clearly defined, valid, reliable, and implemented consistently across all study participants?	Were the outcome assessors blinded to the exposure status of participants?	Was loss to follow-up after baseline 20% or less?	Were key potential confounding variables measured and adjusted statistically for their impact on the relationship between exposure(s) and outcome(s)?	Overall assessment
Arango et al. (2018) [43]	Yes	Yes	NR	Yes	No	Yes	Yes	Yes	Yes	No	Yes	NR	No	No	Fair
Arora et al. (2017) [15]	Yes	Yes	Yes	Yes	No	Yes	Yes	Yes	No	No	Yes	NR	No	Yes	Good
Benner et al. (2017) [44]	Yes	Yes	Yes	Yes	No	Yes	Yes	Yes	Yes	Yes	Yes	NR	NR	Yes	Good
Boen et al. (2020) [34]	Yes	No	Yes	Yes	No	Yes	Yes	Yes	No	No	Yes	NR	Yes	Yes	Good
Cristini et al. (2012) [52]	Yes	No	NR	NR	No	Yes	Yes	Yes	No	No	No	NR	Yes	No	Poor
Davis et al. (2019) [61]	Yes	Yes	Yes	Yes	No	Yes	Yes	Yes	No	Yes	Yes	NR	CD	Yes	Good
DeWit et al. (2011) [53]	Yes	Yes	Yes	Yes	No	Yes	Yes	Yes	Yes	Yes	Yes	NR	Yes	Yes	Good
Fulco et al. (2019) [16]	Yes	Yes	CD	Yes	No	Yes	Yes	Yes	No	Yes	Yes	NR	CD	Yes	Good
Gonzales et al. (2014) [54]	Yes	Yes	Yes	Yes	No	Yes	Yes	Yes	Yes	Yes	Yes	NR	Yes	Yes	Good
Gunnarsódttir et al. (2021) [62]	Yes	Yes	Yes	Yes	No	Yes	Yes	Yes	No	No	No	NR	Yes	No	Good
Hatchel et al. (2018) [55]	Yes	Yes	NR	Yes	No	Yes	Yes	Yes	Yes	Yes	Yes	NR	NR	No	Fair
Jiang et al. (2020) [45]	Yes	Yes	Yes	Yes	No	Yes	Yes	Yes	No	No	Yes	NR	NR	No	Fair
Joyce (2019) [35]	Yes	Yes	NR	Yes	No	Yes	Yes	Yes	No	No	Yes	NR	No	No	Fair
Klinck et al. (2020) [46]	Yes	Yes	Yes	Yes	No	Yes	Yes	Yes	Yes	Yes	Yes	NR	Yes	Yes	Good
Leonard et al. (2016a) [30]	Yes	Yes	NA	Yes	No	Yes	Yes	Yes	No	Yes	Yes	NR	No	Yes	Good
Leonard et al. (2016b) [31]	Yes	Yes	NA	Yes	No	Yes	Yes	Yes	No	Yes	Yes	NR	Yes	Yes	Good
Lester et al. (2013) [63]	Yes	Yes	Yes	Yes	No	Yes	Yes	Yes	No	Yes	Yes	NR	Yes	No	Good
Lester & Cross (2015) [56]	Yes	Yes	Yes	Yes	No	Yes	Yes	Yes	No	Yes	Yes	NR	Yes	No	Good
Li & Lerner (2011) [64]	Yes	No	NR	No	No	Yes	Yes	Yes	No	Yes	Yes	NR	No	Yes	Fair
Loukas et al. (20161) [57]	Yes	Yes	Yes	Yes	No	Yes	Yes	Yes	Yes	Yes	Yes	NR	No	No	Good
Markowitz (2016) [36]	Yes	Yes	NR	Yes	No	Yes	Yes	Yes	No	No	No	NR	No	Yes	Good
McNeil et al. (2020) [32]	Yes	Yes	NA	Yes	No	Yes	Yes	Yes	No	Yes	Yes	NR	Yes	Yes	Good
Moffa et al. (2016) [47]	Yes	Yes	Yes	Yes	Yes	Yes	Yes	Yes	No	No	No	NR	No	No	Fair
Okado et al. (2018) [58]	Yes	Yes	Yes	Yes	No	Yes	Yes	Yes	Yes	No	Yes	NR	Yes	No	Good
Pierre et al. (2020) [48]	Yes	Yes	Yes	Yes	No	Yes	Yes	Yes	Yes	Yes	Yes	NR	NR	No	Good
Pössel et al. (2016) [66]	Yes	Yes	Yes	Yes	No	Yes	Yes	Yes	No	Yes	Yes	NR	No	Yes	Good
Sanders et al. (2020) [49]	Yes	Yes	Yes	Yes	Yes	Yes	Yes	Yes	Yes	Yes	Yes	NR	Yes	No	Good
Shochet & Smith (2014) [59]	Yes	Yes	Yes	Yes	No	Yes	Yes	Yes	Yes	Yes	Yes	NR	Yes	No	Good
Shochet et al. (2011) [60]	Yes	Yes	Yes	Yes	No	Yes	Yes	Yes	Yes	Yes	Yes	NR	Yes	No	Good
Stiles & Gudiño (2018) [33]	Yes	Yes	NA	Yes	No	Yes	Yes	Yes	No	Yes	Yes	NR	NR	No	Fair
Tucker et al. (2011) [50]	Yes	Yes	NR	Yes	No	Yes	Yes	Yes	No	No	Yes	NR	No	Yes	Good
Wickrama & Vazsonyi (2011) [37]	Yes	No	NR	Yes	No	Yes	Yes	Yes	No	No	No	NR	No	No	Poor
Wright & Wachs (2019) [51]	Yes	Yes	Yes	Yes	No	Yes	Yes	Yes	No	No	Yes	NR	Yes	No	Good
Yu et al. (2016) [65]	Yes	Yes	NR	Yes	No	Yes	Yes	Yes	Yes	Yes	Yes	NR	Yes	Yes	Good

*NR * Not Reported, *NA*  Not Applicable, *CD*  Cannot Determine

**Table 4:** Risk of bias assessment for intervention studies

Author (Year)	Was the study described as randomized, a randomized trial, a randomized clinical trial, or an RCT?	Was the method of randomization adequate (i.e., use of randomly generated assignment)?	Was the treatment allocation concealed (so that assignments could not be predicted)?	Were study participants and providers blinded to treatment group assignment?	Were the people assessing the outcomes blinded to the participants' group assignments?	Were the groups similar at baseline on important characteristics that could affect outcomes (e.g., demographics, risk factors, co-morbid conditions)?	Was the overall drop-out rate from the study at endpoint 20% or lower of the number allocated to treatment?	Was the differential drop-out rate (between treatment groups) at endpoint 15 percentage points or lower?	Was there high adherence to the intervention protocols for each treatment group?	Were other interventions avoided or similar in the groups (e.g., similar background treatments)?	Were outcomes assessed using valid and reliable measures, implemented consistently across all study participants?	Did the authors report that the sample size was sufficiently large to be able to detect a difference in the main outcome between groups with at least 80% power?	Were outcomes reported or subgroups analyzed prespecified (i.e., identified before analyses were conducted)?	Were all randomized participants analyzed in the group to which they were originally assigned, i.e., did they use an intention-to-treat analysis?	Quality Rating
Blossom et al. (2020) [41]	Yes	Yes	Yes	NR	NR	Yes	Yes	Yes	NR	No	Yes	No	Yes	NR	Good
Singla et al. (2021) [42]	Yes	Yes	Yes	Yes	Yes	Yes	Yes	Yes	NR	Yes	Yes	No	Yes	NR	Good

### Results of synthesis

As the pattern of findings did not change according to whether a study examined school connectedness using a holistic measure or separate components, our synthesis of results considers ‘school connectedness’ as a single construct, notwithstanding the variation in conceptualisation and measurement described above. Similarly, given that we did not find a discernible pattern for the effect of age or schooling stage on the relationship between school connectedness and depression and anxiety, we have not separated findings by these groupings.

#### Evidence for a protective relationship

Nineteen longitudinal studies found a significant protective relationship between school connectedness and mental health outcomes of interest. These included 15 studies [16, 35, 36, 43, 45, 51, 55, 57, 59, 64] that assessed depressive symptoms (five [34, 37, 44, 50, 62] of which examined school disconnectedness or reductions in school connectedness), one study [51] that assessed anxiety symptoms, and six studies [30, 49, 54, 58, 65] that assessed combined depression/anxiety symptoms (one [52] of which examined school disconnectedness). That is, higher levels of school connectedness predicted lower levels of depressive and/or anxiety symptoms at a later point (noting that the inverse relationship was significant for school disconnectedness). Effects were evident at six-months to five-years follow-up, on average.

Both intervention studies were of ‘good’ quality and showed a significant protective relationship between school connectedness and depressive symptoms. However, Blossom et al. [41] found that school attachment only mediated the effect of the intervention on depressive symptoms approximately 1.5 years later in a sequential mediation model through improvements in self-esteem (indirect effect [95% CI = -0.02 to -0.0005]). After accounting for self-esteem, the direct effect of the intervention on the relationship between school attachment and depression was not significant. Singla et al. [42] found that the effect of the intervention on depression was mediated by improvements in school climate (school climate accounted for 17.8% of the total direct effect on depressive symptoms). When individual school climate components were examined, “relationships at school” at 8 months post-randomisation was significantly associated with less depression at 17 months (51.4% of the total indirect effect of school climate on depressive symptoms), but without an association with “school belonging”.

#### Evidence for risk relationship

One longitudinal study [47] found a significant risk relationship where greater school connectedness predicted higher levels of internal distress (*p* = 0.010), but the effect size was negligible (Cohen’s f^2^ = 0.006) [47].

#### Evidence for a null relationship

Three longitudinal studies [31, 33, 48] from the USA found a non-significant relationship between school connectedness and depression and/or anxiety. Stiles and Gudiño [33] found that for young people in contact with child welfare services, school connectedness did not predict internalising problems one and a half, and three years later. Similarly, Leonard and Gudiño [31] who drew a smaller subsample of participants and measures from the same survey database also found that school connectedness did not predict internalising problems. Pierre et al. [48] found a non-significant relationship between school connectedness and depression, anxiety and stress approximately one year later in a sample of African American males.

#### Studies with mixed results

Nine studies [15, 32, 46, 53, 56, 60, 61, 63, 66] reported mixed results, of which all examined depression, three [46, 53, 56] examined anxiety, and one [56] examined combined depression/anxiety. Across all studies with mixed results, approximately half of all reported associations were protective and approximately half were not significant. Only two studies reported a risk relationship among their results [53, 66]. However, all but one of the studies [66] concluded that there was a significant protective effect for school connectedness on the mental health outcomes of interest.

Studies reported mixed results due to differences according to sex/gender (see below) [46, 60, 61, 63, 66]; for various components of school connectedness that were included in the analysis [15, 56, 60] (e.g., a significant protective effect for teacher support on depression but not for school engagement) [15]; across mental health outcomes (e.g., a significant protective effect for depression but not anxiety) [46]; and owing to the study design and statistical models [32, 53, 63, 66] (e.g., a significant risk relationship between the intercept and slope but a non-significant relationship between the slope and slope, for the same variables in the same model) [66].

#### How does school connectedness predict depression and anxiety?

We did not identify any longitudinal studies that examined potential mediators of the association between school connectedness and anxiety and depression. However, we identified five studies which examined school connectedness as a mediator [35, 45, 54, 55, 59]. For example, Hatchel et al. [55] found that school belonging mediated the relationship between victimization and depression. Similarly, Jiang et al. [45] found that emotional school engagement (e.g., “My class has a good atmosphere”, “I feel close to people in this school”) partially mediated the relationship between teacher discrimination and depression.

#### For whom does school connectedness predict depression and anxiety?

Seven longitudinal studies [16, 36, 46, 60, 61, 63, 66] examined potential differences in effects between sex/genders but no discernible pattern of sex/gender differences was identified. For example, Davis et al. [61] found a protective association in females but a non-significant association within the whole sample and Klinck et al. [46] found stronger effects in females, whereas Fulco et al. [16] and Lester et al. [63] found protective relationships in both males and females at different ages and Markowitz et al. [36] found that low school connectedness was a risk factor only in males who had experienced early adversity. One additional study [48] was conducted with African American males only and reported null effects.

Four longitudinal studies [32, 37, 46, 51] conducted in the USA found moderation or interaction effects for minority groups. For example, Klinck et al. [46] found higher levels of school connectedness at baseline were associated with lower depression at follow-up for adolescents identifying as non-Hispanic White, Hispanic, or Latinx, but not for adolescents identifying as Black/African American. Wickrama and Vazsoni [37] found an interaction effect such that school disengagement had a stronger influence on changes in depressive symptoms for Hispanic American adolescents than for European American adolescents.

Two longitudinal studies [15, 46] found an interaction effect between anxiety, depression, and school connectedness. Klinck et al. [46] found that more school connectedness at baseline significantly predicted less depression at follow-up only in adolescents at low risk of an anxiety disorder at baseline. The relationship was not significant in adolescents at high risk of an anxiety disorder. In contrast, Arora et al. [15] found that high levels of anxiety at baseline were associated with increased levels of depressive symptoms at follow-up, but this association was only significant when teacher support was moderate-to-high at baseline, not under conditions of low teacher support.

## Discussion

Growing evidence of rising rates of student mental disorder [1] and knowledge of the extent to which student depression and anxiety contribute to poor learning outcomes [67, 68] are reorientating both schools and the health sector towards the understanding that schools are communities that are relationally rich, and which can affect both mental health and learning [8]. This systematic review of the evidence for relationships between school connectedness and depression and anxiety from longitudinal and intervention studies showed an overall pattern of results that overwhelmingly indicated that higher levels of school connectedness predict lower levels of depressive and anxiety symptoms in young people in secondary school. There were notably fewer longitudinal studies and no intervention studies examining anxiety symptoms alone, despite anxiety being the most common mental health problem experienced by young people [69]. Although we only identified two intervention studies, the evidence from both for depressive symptoms was promising with significant effects around one-and-half-years post-intervention. We were unable to determine the extent to which improvement of school connectedness plays a role in the remission of depression and anxiety as, with the exception of one intervention study [41], no other studies intentionally recruited samples with existing depression and anxiety. No studies were conducted during the COVID-19 pandemic.

These findings are consistent with previous cross-sectional studies showing that greater school connectedness is associated with better mental health [19, 40]. They are also consistent with the experiences of our youth advisers who described the importance of school connectedness for mental health. As one youth adviser, 18, from Australia reflected, *“I've had mental health issues my whole life… I noticed the second that I moved schools to a more healthy environment, the rapid improvement of my mental health.”* Another youth adviser, 18, from Indonesia explained, *“Knowing your school is there for you really calms you down, takes one more thought out of your head, and more weight off your shoulders,”* while another, 21, from the Philippines, described school as a *“second home”.* Our findings indicate that very few interventions that were designed to improve school connectedness assessed depression and anxiety outcomes, in much the same way that school-based interventions designed to improve health typically fail to include educational outcomes [70]. This highlights an important opportunity for inter-sectoral collaboration between mental health and education researchers.

We identified a smaller number of studies which reported null effects, and even fewer that reported a risk relationship. These differences in reported results may be explained by the heterogeneity between studies. Critically, there was wide variation in how school connectedness was defined, measured, and analysed. Some studies treated school connectedness as a multifaceted construct that was analysed using a total score, while others analysed how specific components of school connectedness (e.g., peer support, teacher relationships, engagement with learning) related to depression or anxiety outcomes. While the notion that school connectedness is a multifaceted construct has been widely reported [13, 14] and was reinforced by our youth advisers (see Table 5), this variation makes comparisons between studies challenging and it is difficult to determine which components of school connectedness are driving the effects. Further, it may be that school connectedness has a stronger association with depression and anxiety in some individuals but not others. For example, we found a small body of evidence around the moderating effect of race/ethnicity and levels of comorbid anxiety [15, 32, 37, 46, 51]. Several individual (e.g., gender, age, comorbid diagnoses, personality) and contextual (e.g., friends outside of school, relationships with family members, exposure to discrimination and bullying, geographical location, school characteristics, cultural practices) factors may contribute to a person’s experience of school connectedness and depression and anxiety [19, 71], which were not necessarily assessed in the included studies. As one youth adviser, 16, explained, ***“****In Indonesia you can't really dismiss religion. You can't ignore it because it's so deeply rooted in our society and that in turn reflects [on] other things like our mental health and even school connectedness.”*

**Table 5:** Reflections from youth advisers about the construct of school connectedness

The conceptualisation of school connectedness as a multifaceted construct comprising both relational or social aspects in addition to engaging with the wider environment of a school and learning experiences was reinforced by our youth advisers. Youth advisers shared that school connectedness encompasses notions of: feeling acknowledged by teachers, peers, parents and the wider school community; relationships characterised by empathy, care, active communication, respect, and genuineness; a cohesive and welcoming school environment; feeling included, a sense of belonging and not feeling alone; feeling able to express your identity and personal strengths; and engaging in learning and participating in enjoyable school activities. As one youth adviser, 16, from Australia explained:
*“You've got that social aspect, but you've also got extra-curricular activities, how you're going through your studies, your classes, if you're enjoying them, it’s engagement… being supported in all aspects of your wellbeing, it's the positive emotions, it's the relationships, it's the meaning, it’s engagement, the accomplishment, it's all of that. Once you feel supported in all these areas is when you feel connected… It's hard to define it as one thing… and if we want to measure it, we have to measure different areas.”*
The relational components of school connectedness were considered paramount; even when youth advisers felt connected to the school as an institution and enjoyed engaging in activities and learning, poor relationships with teachers, peers and other school staff had a strong impact on overall sense of connectedness. They reported that the quality, rather than the quantity, of relationships was critical. One youth adviser, 18, from Indonesia highlighted the importance of this by saying:
*“If I had all the money in the world… it would be that everyone in the school really cares about their students, they know their interests, and their names, and every time they talk about something they just connect in a really genuine way.”*

Taken together, these findings fill an important gap in the evidence base and suggest that improving school connectedness may be a novel intervention target for the prevention of depression and anxiety. While more studies conducted beyond the USA and in a range of schooling systems (e.g., public, private, tertiary) are needed, it is noteworthy that interventions designed to improve school connectedness were feasible and effective at improving depressive symptoms in both high-income [41] and low-middle income countries [42]. However, several limitations of the review evidence should be acknowledged and addressed, primarily related to the methodology of the included studies. Notably, measures of school connectedness were rarely validated, and their psychometric properties were often not reported. There was also inconsistent and incomplete reporting of effect sizes and estimates of uncertainty required for meta-analysis to quantify the strength of the protective effect. As many studies did not report participants lost to follow-up, attrition bias may contribute to overstating the protective effect or bias findings towards a specific group (e.g., those who have stayed in school rather than dropped out). The failure of some studies to adjust for key confounders such as age, sex/gender, and SES also limits causal inferences.

With these considerations in mind, understanding how school connectedness changes over time and how this relates to the emergence of depression and anxiety within the wider context of young people’s development context (e.g., pubertal changes, changes in family relationships, orientation to peers, and transitions from primary to secondary school, or secondary to tertiary schooling) will be an important avenue for future prospective studies to inform the design and timing of delivery of interventions. School connectedness is likely to be a developmental process, which begins prior to primary school, is affected by various elements of the school experience (e.g., peer relationships, parental involvement, number of schools attended) and student factors (e.g., levels of literacy, social anxiety), and has a cumulative impact on student outcomes over time (e.g.,[11, 12]). This suggests that improving school connectedness needs to occur at all ages, appreciating that strategies for improvement must be developmentally appropriate and may have greater potency at particularly sensitive periods. Interestingly, both intervention studies were conducted with young people approximately 13–14 years old. Combined with evidence of the increase in incidence of anxiety and depression around this age [72, 73] and emerging evidence that the transition from primary to secondary school is a particularly vulnerable time for experiencing disconnection from school and learning [11, 12], the effectiveness of school connectedness interventions delivered in early secondary school on concurrent and later anxiety and depression may be particularly strong.

There are likely to be multiple mechanisms underpinning the relationship between school connectedness and depression and anxiety such as relationship quality, levels of motivation, feelings of loneliness, sense of purpose, academic pressure, and social expectations of behaviour. This review revealed a gap in this evidence base. Identifying specific mechanisms will be important for targeting intervention strategies more effectively and will also assist in understanding differences in protection and risk between individuals. For example, the level of academic or social pressure experienced by students may be an important mediator in understanding why more school connectedness predicts higher levels of (social) anxiety in some individuals [47, 53]. While this risk relationship is not well-established in the existing literature, it resonated with our youth advisers and warrants further exploration. As one youth adviser from Australia, 18, described, *“The more I was connected to school, the worse my mental health got because there was a lot of pressure in trying to maintain those connections. I had to act a certain way, talk a certain way…”,* while another youth adviser, 16, reflected*, “Our expectations of the perfect student … needs to change … teachers really need to take on the fact that all students are not the same, they don't fit in the same box…”.*

Finally, we need more intervention studies that assess depression and anxiety outcomes over long follow-up periods to determine the persistence of effects. Studies should also assess diagnoses and remission status in young people already experiencing clinically significant symptoms, in addition to educational outcomes and broader measures of social-emotional wellbeing (e.g., emotional regulation, interpersonal skills, resilience). Indeed, multisectoral interventions may need to demonstrate benefits to both the health and education sectors to be sustained and scaled. The cost-effectiveness of these interventions is yet to be evaluated, from either the perspective of the health or education sectors. Given increasing enrolment and retention of young people in schooling worldwide [5, 6], interventions to promote school connectedness are likely to be highly accessible and scalable. Our youth advisers shared several strategies for how school connectedness is successfully embedded in their day-to-day school life (e.g., parent-teacher-student conferences to discuss progress and goals beyond academics, a buddy system, offering new activities including during remote learning, student representatives at assembly) which they viewed positively, suggesting the acceptability of school connectedness interventions for young people.

The strengths of this review were its broad search strategy including various terms associated with school connectedness and depression and anxiety and engagement with youth advisers with lived experience. Despite the inclusion of tertiary education settings in the search strategy, no studies were identified, which precludes further comment, notwithstanding expectations of relevance. Due to heterogeneity between studies, we were not able to conduct a meta-analysis. No studies examined mediators of the association between school connectedness and depression and anxiety outcomes which limits our recommendations. While the quality of studies was generally good, we retained studies rated as ‘poor’ and ‘fair’ in the synthesis of the results and it was not possible to assess publication bias. Due to resourcing constraints, we did not conduct blinded, independent article screening, data extraction, and study quality assessment with two researchers. No studies were conducted within the COVID-19 pandemic during which multiple challenges in maintaining school connectedness during virtual learning [74] and in the return to onsite learning [75] have been described. These experiences have powerfully enhanced community awareness of the importance of schools, not just as places of learning, but as social communities through which health and wellbeing emerge. This suggests that examining the complexities of the pandemic’s effect on school connectedness and mental health will be important to consider in future work. This will require consideration of potential benefits as well as harms, differences across contexts, and the need to ensure that prevention-oriented interventions remain in focus, notwithstanding the pressures faced by schools to respond to students with acute emotional distress.

## Conclusions

School connectedness moves beyond individual-level and academic factors to recognise the profound effects of young people’s social-emotional environments on mental health, which in turn can benefit learning. Accessible to both health and education sectors, preventive interventions that target school connectedness have the potential to be scalable, with the ability to reach large numbers of young people, including in LMICs where secondary education systems are rapidly expanding. Consistent with global policy [76], promoting school connectedness may be a good investment to promote student mental health and prevent mental disorder.

## Supplementary Information

**Additional file 1.** MEDLINE Search; date searched July 12^th^, 2021.

## Data Availability

All data analysed in this study are secondary (retrieved from original studies included in the review) and are included in this published article (and its additional information files). Other data generated in this study are available from the corresponding author on reasonable request.

## Abbreviations

CDI: Children’s Depression Inventory
CESD: Center for Epidemiological Studies – Depression scale
COVID-19: Coronavirus disease
DASS-21: Depression Anxiety Stress Scale
HIC: High-income country
HSTP: The High School Transition Program
LMIC: Low- and middle-income country
NSCAW: National Survey of Child and Adolescent Well-Being
SEHER: Strengthening Evidence base on scHool-based intErventions for pRomoting adolescent health program
USA: United States of America
