# Supplementary material for: The role of school connectedness in the prevention of youth depression and anxiety: a systematic review with youth consultation
Source: BMC Public Health. 2022 Nov 25;22:2152. doi: 10.1186/s12889-022-14364-6 (PMC9694921; doi:10.1186/s12889-022-14364-6)
Supplement: Supplementary file 1 — Additional file 1. MEDLINE Search; date searched July 12th, 2021. [file 12889_2022_14364_MOESM1_ESM.docx]

# Additional File 1

## MEDLINE Search; date searched July 12^th^, 2021

| 1 | ((school* adj2 connect*) or (school* adj2 disconnect*) or (teacher* adj2 connect*) or (peer* adj2 connect*) or (connect* adj2 learning) or (school* adj2 belong*) or (school* adj2 identification) or (school* adj2 affiliat*) or (school* adj2 membership) or (school* adj2 attachment) or school-bond* or ((connect* adj2 adult*) and (at-school? or in-school?)) or (relation* adj2 school*) or (participat* adj2 school?) or (school* adj2 engag*) or (school* adj2 disengag*) or (school* adj3 climate) or (school adj2 experience*)).tw,kf. |
| --- | --- |
| 2 | (boy or boys or girl or girls or child or children or childhood or adolescen* or pediatric* or paediatric* or youth or youths or teen or teens or teenage* or school-age* or schoolage* or school-child* or schoolchild* or school-girl* or schoolgirl* or school-boy* or schoolboy* or student* or ((young or emerging) and (adult* or people or person or persons)) or AYA or AYAs).af. |
| 3 | *affective symptoms/ or *depression/ or *mood disorders/ or *depressive disorder/ or *depressive disorder, major/ or *depressive disorder, treatment-resistant/ or *dysthymic disorder/ or *premenstrual dysphoric disorder/ or *seasonal affective disorder/ |
| 4 | (affective-symptom* or affective-disorder* or depress* or mood-disorder* or dysthymic-disorder* or dysthymia* or premenstrual-dysphoric-disorder* or seasonal-affective-disorder*).tw,kf. |
| 5 | *anxiety disorders/ or *agoraphobia/ or *anxiety, separation/ or *panic disorder/ or *phobic disorders/ or *phobia, social/ or *anxiety/ or *catastrophization/ or *psychological distress/ |
| 6 | (anxiety or agoraphobia* or panic-disorder* or phobic-disorder* or phobia* or psychological-distress or emotional-distress or mental-distress).tw,kf. |
| 7 | *mental health/ |
| 8 | (common-mental-disorder* or emotional-problem* or (internali* adj symptom*) or (internali* adj disorder*) or (externali* adj symptom*) or (externali* adj disorder*) or mental-health or emotional-health or psychological-health or mental-wellbeing or psychological-wellbeing or emotional-wellbeing or mental-well-being or psychological-well-being or emotional-well-being).tw,kf. |
| 9 | 1 and 2 and (3 or 4 or 5 or 6 or 7 or 8) |
| 10 | limit 9 to (english language and yr="2011 -Current") |
| 11 | limit 10 to (case reports or comment or editorial or guideline or letter or practice guideline) |
| 12 | limit 10 to "review" |
| 13 | limit 10 to "systematic review" |
| 14 | 12 not 13 |
| 15 | 10 not (11 or 14) |
